# Supplementary material for: Spatial Variation in Stream-Water Bacterial Communities Across Sites Differing in Wild Chinese Giant Salamander Detection Frequency
Source: Biology (Basel). 2026 Sep 18;15(18):1647. doi: 10.3390/biology15181647 (PMC13604818; doi:10.3390/biology15181647)
Supplement: Supplementary file 1 [file biology-15-01647-s001.zip › Data Related to “Spatial Variation in Stream-Water Bacterial Communities across Sites Differing in Wild Chinese Giant Salamander Detection Frequency”/Microbial Data/report/src/hyperlinks/ReadMe_qiime2_LRS_EN.html]

| result |  |
| --- | --- |
| \|-- 01.CleanData | 【Quality-controlled tags information】 |
| \| \|-- data\_table.xls | 【Statistical form for reads merging process of all samples】 |
| \| \|-- Sample\_Name | 【Results of quality control for each sample】 |
| \| \| \|-- \*.fna.gz | 【Clean tags (FASTA format)】 |
| \| \| \|-- \*.fastq.gz | 【Clean tags (FASTQ format)】 |
| \| \| \|-- \*.withprimer.fastq.gz | 【Clean tags with primer (FASTQ format)】 |
| \|-- 02.FeatureAnalysis | 【Feature sequences and tables】 |
| \| \|-- aligned-rep-seqs.qza | 【Aligned with mutli-seqs】 |
| \| \|-- denoisingTable\_qza | 【ASVs abundance denosized (qza viewable file)】 |
| \| \|-- denoisingTable.qza | 【ASVs abundance denosized (qza format)】 |
| \| \|-- denoisingTable\_qzv | 【ASVs abundance denosized (qzv viewable file)】 |
| \| \|-- denoisingTable.qzv | 【ASVs abundance denosized (qzv format)】 |
| \| \|-- feature.fasta | 【Feature sequences】 |
| \| \|-- featureSeqs\_qza | 【Feature sequences (qza viewable file)】 |
| \| \|-- featureSeqs.qza | 【Feature sequences (qza format)】 |
| \| \|-- featureSeqs\_qzv | 【Feature sequences (qzv viewable file)】 |
| \| \|-- featureSeqs.qzv | 【Feature sequences (qzv format)】 |
| \| \|-- featureTable\_qza | 【Feature tables for abundance (qza viewable file)】 |
| \| \|-- featureTable.qza | 【Feature tables for abundance (qza format)】 |
| \| \|-- featureTable\_qzv | 【Feature tables for abundance (qzv viewable file)】 |
| \| \|-- featureTable.qzv | 【Feature tables for abundance (qzv format)】 |
| \| \|-- feature.tax\_assignments.txt | 【The table of taxonomy annotation】 |
| \| \|-- masked-aligned-rep-seqs.qza | 【Multiple sequence alignment results after filtering high variability regions】 |
| \| \|-- rooted-tree\_qza | 【Rooted tree (qza viewable file)】 |
| \| \|-- rooted-tree.qza | 【Rooted tree (qza format)】 |
| \| \|-- unrooted-tree\_qza | 【Unrooted (qzv viewable file)】 |
| \| \|-- unrooted-tree.qza | 【Unrooted tree (qzv format)】 |
| \| \|-- seq\_taxonomy\_qza | 【Taxonomy annotation results (qza viewable file)】 |
| \| \|-- seq\_taxonomy.qza | 【Taxonomy annotation results (qza format)】 |
| \| \|-- seq\_taxonomy\_qzv | 【Taxonomy annotation results (qzv viewable file)】 |
| \| \|-- seq\_taxonomy.qzv | 【Taxonomy annotation results (qzv format)】 |
| \| \|-- tables | 【Feature tables】 |
| \| \| \|-- sample/group1/... | 【Feature tables of (sample, group)】 |
| \| \| \| \|-- featureTable\_Absolute | 【Feature tables for absolute abundance】 |
| \| \| \| \| \|-- featureTable.\*.\*.absolute.txt | 【Absolute abundance summarized at each taxonomic level (total,k,p,c,o,f,g,s)】 |
| \| \| \| \|-- featureTable\_Relative | 【Feature tables for relative abundance】 |
| \| \| \| \| \|-- featureTable.\*.\*.relative.xls | 【Relative abundance summarized at each taxonomic level (total,k,p,c,o,f,g,s)】 |
| \| \| \| \|-- featureTable\_Even | 【Feature tables for absolute abundance after normalization（The sum of each OTU/ASV abundance of each sample is equal)】 |
| \| \| \| \| \|-- featureTable.\*.total.even.txt | 【Absolute abundance after normalization】 |
| \| \| \| \| \|-- featureTable.\*.\*.absolute.xls | 【Absolute abundance summarized after normalization at each taxonomic level (total,k,p,c,o,f,g,s)】 |
| \| \| \| \|-- featureTable\_Biom | 【Feature tables (Biom format)】 |
| \| \| \| \| \|-- featureTable.\*.total.absolute.txt.biom | 【Absolute abundance (Biom format)】 |
| \| \| \| \| \|-- featureTable.\*.total.even.txt.biom | 【Absolute abundance after normalization (Biom format)】 |
| \| \| \| \| \|-- featureTable.\*.total.relative.txt.biom | 【Relative abundance after normalization (Biom format)】 |
| \| \| \| \|-- featureTable\_tableStat | 【Taxonomy annotation results for (sample, group)】 |
| \| \| \| \| \|-- featureTable.briefStatInfo.txt | 【Taxonomy annotation brief results for (sample, group)】 |
| \| \| \| \| \|-- featureTable.relativeStat.\*.xls | 【Taxonomy annotation results for (sample, group) at each taxonomic level (k,p,c,o,f,g,s)】 |
| \| \| \| \| \|-- classifiedStat.sample.absolute.{xls,svg,png} | 【Annotated statistical results and visual display at each taxonomic level (k,p,c,o,f,g,s)】 |
| \| \| \| \| \|-- featureTable.\*.Tags\_stat.xls | 【Statistical table of tags number for (sample, group)】 |
| \| \| \| \| \|-- featureTable.\*.TagsDistribution.{svg,,png}} | 【Statistical table of tags number and visual display】 |
| \|-- 03.TaxonVisual | 【Visualization results of taxonomy annotation】 |
| \| \|-- barplot | 【Species abundance layout】 |
| \| \| \|-- sample/group1/... | 【The barplot of tax relative abundance for (sample, group)】 |
| \| \| \| \|-- top10.\*.\*.barplot.{xls,png,svg} | 【Top 10 taxonomy relative abundance layout at each taxonomic level for sample】 |
| \| \| \| \|-- top30.\*.g.barplot.{xls,png,svg} | 【Top 30 taxonomy relative abundance layout at genus level for sample】 |
| \| \|-- heatmap | 【Heatmap result】 |
| \| \| \|-- sample/group1/... | 【Heatmap of clustering of taxonomy annotation】 |
| \| \| \| \|-- heatmap.\*.\*.{pdf,png,txt} | 【Species abundance heatmap at taxonomic levels (phylum,class,family,genus)】 |
| \| \|-- tree100 | 【GraPhlan of tree100】 |
| \| \| \|-- sample/group1/... | 【GraPhlan for (sample, group)】 |
| \| \| \| \|-- genus\_100.tree | 【Phylogenetic tree for top 100 genus】 |
| \| \| \| \|-- genus\_100.tree.{png,svg} | 【Phylogenetic tree and visual display】 |
| \| \|-- venn | 【Venn diagram】 |
| \| \| \|-- sample/group1/... | 【Venn diagram for (sample, group)】 |
| \| \| \| \|-- \* | 【Date of (sample, group) for venn diagram analysis, e.g. A-B】 |
| \| \| \| \| \|-- \*\_\*.txt | 【Characteristic features for venn diagram, e.g. A-B\_A.txt】 |
| \| \| \| \| \|-- \*\_\*:\*.txt | 【Common features for venn diagram, e.g. A-B\_A：B.txt】 |
| \| \| \| \| \|-- \*.{png,svg} | 【Venn diagram for samples/groups】 |
| \| \|-- flower | 【Flower diagram】 |
| \| \| \|-- sample/group1/... | 【Flower diagram for (sample, group)】 |
| \| \| \| \|-- \* | 【Date of (sample, group) for flower diagram analysis, e.g. A-B-C-D-E-F】 |
| \| \| \| \| \|-- \*\_all-core.xls | 【Common features for flower diagram, e.g. A-B-C-D-E-F\_all-core.xls】 |
| \| \| \| \| \|-- \*\_\*.xls | 【Characteristic features for flower diagram, e,g, A-B-C-D-E-F\_A.xls】 |
| \| \| \| \| \|-- \*.{xls,png,svg} | 【Flower diagram for samples/groups】 |
| \| \|-- ternary | 【Ternaryplot analysis】 |
| \| \| \|-- sample/group1/... | 【Ternaryplot analysis among 3 samples(or groups)】 |
| \| \| \| \|-- \*/\*.ternary.{jpeg,pdf} | 【Ternaryplot analysis results for each Comparison at each taxonomic level】 |
| \|-- 04.AlphaDiversity | 【Alpha Diversity】 |
| \| \|-- alpha\_index\_table | 【Alpha diversity indices】 |
| \| \| \|-- sample/group1/... | 【Alpha diversity indices of samples and groups】 |
| \| \| \| \|-- alpha\_diversity\_index.{sample,group}.txt | 【Tables of alpha diversity indices】 |
| \| \|-- visual\_specaccum | 【Visual specaccum result】 |
| \| \| \|-- sample | 【Visual specaccum curve】 |
| \| \| \| \|-- specaccum\_test.{pdf,png} | 【Visual specaccum curve for samples/groups】 |
| \| \|-- visual\_rankAbundance | 【Rank Abundance result】 |
| \| \| \|-- sample/group1/... | 【Pictures of rank abundance curve】 |
| \| \| \| \|-- rank\_abundance.{sample,group}.{pdf,png} | 【Pictures of rank abundance curve for samples/groups】 |
| \| \|-- visual\_rarefaction | 【Rarefaction curves】 |
| \| \| \|-- sample/group1/... | 【Pictures of rarefaction curves for samples/groups】 |
| \| \| \| \|-- \*/\*.{sample,group}.{pdf,png,xls} | 【Rarefaction curves of(chao1, goods\_coverage, observed\_species, PD\_whole\_tree, shannon,simpson)】 |
| \| \|-- visual\_alphaDiversity | 【Boxplots analysis for alpha diversity indices】 |
| \| \| \|-- group1/... | 【Boxplots analysis for samples/groups】 |
| \| \| \| \|-- \* | 【Boxplots analysis (ACE, chao1, goods\_coverage, observed\_species, PD\_whole\_tree, shannon, simpson)】 |
| \| \| \| \| \|-- \*.{pdf,png} | 【Boxplots analysis of alpha diversity indices】 |
| \| \| \| \| \|-- \*\_kruskalWallis.{txt,pdf,png} | 【KruskalWallis-test of alpha diversity indices (Samples in the group are greater than 2)】 |
| \| \| \| \| \|-- \*\_Tukey.{txt,pdf,png} | 【Tukey-test of alpha diversity indices (Samples in the group are greater than 2)】 |
| \| \| \| \| \|-- \*\_test.{txt,pdf,png} | 【T-test of alpha diversity indices (Samples in the group are greater than 2)】 |
| \| \| \| \| \|-- \*\_wilcox.{txt,pdf,png} | 【Wilcoxon rank sum test of alpha diversity indices (Samples in the group are greater than 2)】 |
| \|-- 05.BetaDiversity | 【Beta diversity analysis】 |
| \| \|-- beta\_distance | 【Beta diversity indices based on analytical distance】 |
| \| \| \|-- sample/group1/... | 【Beta diversity indices for samples/groups】 |
| \| \| \| \|-- \*\_distance\_matrix\_qza | 【(bray\_curtis,jaccard,(un)weighted\_unifrac) Distance matrix (qza viewable file)】 |
| \| \| \| \|-- \*\_distance\_matrix.qza | 【(bray\_curtis,jaccard,(un)weighted\_unifrac) Distance matrix (qza format)】 |
| \| \| \| \|-- \*\_pcoa\_results\_qza | 【(bray\_curtis,jaccard,(un)weighted\_unifrac) PCoA (qza viewable file)】 |
| \| \| \| \|-- \*\_pcoa\_results.qza | 【(bray\_curtis,jaccard,(un)weighted\_unifrac) PCoA (qza format)】 |
| \| \|-- beta\_upgma | 【Beta UPGMA analysis】 |
| \| \| \|-- sample/group1/... | 【UPGMA analysis for samples/groups】 |
| \| \| \| \|-- (un)weighted\_unifrac | 【UPGMA analysis based on (un)weigted unifrac distances】 |
| \| \| \| \| \|-- (un)weighted\_unifrac.{sample,group}\_upgma.tre | 【UPGMA tree based on (un)weigted unifrac distances】 |
| \| \| \| \| \|-- upgam.(un)weighted\_unifrac.{sample,group}.tree.{svg,png} | 【UPGMA tree diagram】 |
| \| \|-- beta\_pca | 【Principle Component Analysis (PCA)】 |
| \| \| \|-- sample/group1/... | 【PCA analysis for samples/groups】 |
| \| \| \| \|-- PCA.{xls,pdf,png} | 【PCA results with sample names】 |
| \| \| \| \|-- PCA\_lable.{pdf,png} | 【PCA results without labeling sample names】 |
| \| \| \| \|-- PCA\_circle.{pdf,png} | 【PCA results with sample names with cluster】 |
| \| \| \| \|-- PCA\_circleLable.{pdf,png} | 【PCA results without labeling sample names with cluster】 |
| \| \|-- beta\_pcoa | 【Principal Co-ordinates Analysis (PCoA)】 |
| \| \| \|-- sample/group1/... | 【PCoA analysis for samples/groups】 |
| \| \| \| \|-- PCoA\_display.{pdf,png} | 【PCoA display based on weighted\_unifrac and unweighted\_unifrac distance】 |
| \| \| \| \| \|-- PCoA.{xls,pdf,png} | 【PCoA results with sample names】 |
| \| \| \| \| \|-- PCoA\_label.{pdf,png} | 【PCoA results without labeling sample names】 |
| \| \| \| \| \|-- PCoA\_circle.{pdf,png} | 【PCoA results with sample names with cluster】 |
| \| \| \| \| \|-- PCoA\_circleLabel.{pdf,png} | 【PCoA results without labeling sample names with cluster】 |
| \| \| \| \| \|-- emperor\_pcoa\_plots | 【3D PCoA analysis html format】 |
| \| \|-- beta\_nmds | 【Non-Metric Multi-Dimensional Scaling (NMDS)】 |
| \| \| \|-- sample/group1/... | 【NMDS analysis for samples/groups】 |
| \| \| \| \|-- NMDS\_display.{pdf,png} | 【NMDS display based on weighted\_unifrac and unweighted\_unifrac distance】 |
| \| \| \| \| \|-- \*.NMDS.{xls,pdf,png} | 【NMDS results with sample names】 |
| \| \| \| \| \|-- \*.NMDS\_lable.{pdf,png} | 【NMDS results without labeling sample names】 |
| \| \| \| \| \|-- \*.NMDS\_circle.{pdf,png} | 【NMDS results with sample names with cluster】 |
| \| \| \| \| \|-- \*.NMDS\_circleLable.{pdf,png} | 【NMDS results without labeling sample names with cluster】 |
| \| \|-- beta\_dca | 【Decision Curve Analysis (DCA)】 |
| \| \| \|-- sample/group1/... | 【DCA analysis for samples/groups】 |
| \| \| \| \|-- DCA.{xls,pdf,png} | 【DCA analysis data and diagram】 |
| \| \|-- beat\_unifracHeatmap | 【Heatmap of unifrac distances】 |
| \| \| \|-- sample/group1/... | 【Heatmap of unifrac distances for samples/groups】 |
| \| \| \| \|-- beta\_diversity.heatmap.{svg,png} | 【Heatmap of weighted unifrac distances】 |
| \| \| \| \|-- beta\_diversity.heatmap.(un)Weighted.{svg.png} | 【Heatmap of unweighted unifrac distances】 |
| \| \|-- beta\_div | 【Box plots of Beta Diversity】 |
| \| \| \|-- group1/... | 【Box plots for comparison of Beta Diversity】 |
| \| \| \| \|-- (un)weighted\_unifrac | 【Analysis of beta Diversity indices based on (un)weigted unifrac distances (Samples in the group are greater than 2)】 |
| \| \| \| \| \|-- (un)weighted\_unifrac.{pdf,png} | 【Box plots of Beta Diversity indices based on (un)weigted unifrac distances】 |
| \| \| \| \| \|-- (un)weighted\_unifrac\_kruskalWallis\_all.txt | 【Analysis of variance among groups based on kruskalWallis-test】 |
| \| \| \| \| \|-- (un)weighted\_unifrac\_kruskalWallis.{pdf,png} | 【Visualization of variance among groups based on kruskalWallis-test】 |
| \| \| \| \| \|-- (un)weighted\_unifrac\_TukeyHSD\_all.txt | 【Analysis of variance among groups based on unweighted unifrac distance】 |
| \| \| \| \| \|-- (un)weighted\_unifrac\_TukeyHSD.{pdf,png} | 【Visualization of variance among groups based on unweighted unifrac distance】 |
| \| \| \| \| \|-- (un)weighted\_unifrac\_tTest\_all.txt | 【Analysis of variance among groups based on T-test】 |
| \| \| \| \| \|-- (un)weighted\_unifrac\_tTest.{pdf,png} | 【Visualization of variance among groups based on T-test】 |
| \| \| \| \| \|-- (un)weighted\_unifrac\_twoWilcox\_all.txt | 【Analysis of variance among groups based on twoWilcox】 |
| \| \| \| \| \|-- (un)weighted\_unifrac\_twoWilcox.{pdf,png} | 【Visualization of variance among groups based on twoWilcox】 |
| \|-- 06.StatisticalTest | 【Statistical test】 |
| \| \|-- adonis | 【Permutational Multivariate Analysis of Variance Using Distance Matrices(Adonis)】 |
| \| \| \|-- group1/... | 【Adonis (Samples in the group are greater than 2)】 |
| \| \| \| \|-- bray\_adonis.txt | 【Analysis result of Adonis】 |
| \| \|-- anosim | 【Analysis of Similarity (Anosim)】 |
| \| \| \|-- group1/... | 【Anosim (Samples in the group are greater than 2)】 |
| \| \| \| \|-- stat\_anosim.txt | 【Analysis result of Anosim】 |
| \| \|-- mrpp | 【Multi-response permutation procedure (MRPP)】 |
| \| \| \|-- group1/... | 【MRPP (Samples in the group are greater than 2)】 |
| \| \| \| \|-- stat\_mrpp.txt | 【Analysis result of MRPP】 |
| \| \|-- KruskalWallis | 【KruskalWallis】 |
| \| \| \|-- group1/... | 【KruskalWallis (Samples in the group are greater than 2)】 |
| \| \| \| \|-- phylum/... | 【KruskalWallis analysis at each taxonomic level (total,k,p,c,o,f,g,s)】 |
| \| \| \| \| \|-- \*-\* | 【KruskalWallis for two groups】 |
| \| \| \| \| \| \|-- KruskalWallis\_all.xls | 【All Significantly Different Species Across Groups】 |
| \| \| \| \| \| \|-- KruskalWallis\_sig.xls | 【Differential Species (p < 0.05) Among Groups】 |
| \| \| \| \| \| \|-- KruskalWallis\_dunn.xls | 【Dunn’s Test Results】 |
| \| \| \| \| \| \|-- KruskalWallis\_top\*.xls | 【Top 10 Differential Species by Abundance at Each Taxonomic Level】 |
| \| \| \| \| \| \|-- KruskalWallis\_top\_releative.xls | 【Top 10 Most Abundant Species Table at Each Taxonomic Level】 |
| \| \| \| \| \| \|-- kw\_barplot.{pdf ,png} | 【Mean Abundance Bar Plot Showing Kruskal-Wallis Test Results】 |
| \| \| \| \| \| \|-- kw\_boxplot.{pdf,png} | 【Mean Abundance Boxplot with Kruskal-Wallis Test Results】 |
| \| \|-- Wilcoxon | 【Wilcoxon analysis】 |
| \| \| \|-- group1/... | 【Wilcoxon (Samples in the group are greater than 2)】 |
| \| \| \| \|-- phylum/... | 【Wilcoxon analysis at each taxonomic level (total,k,p,c,o,f,g,s)】 |
| \| \| \| \| \|-- \*-\* | 【Wilcoxon for two groups】 |
| \| \| \| \| \| \|-- Wilcoxon\_all.xls | 【All Significantly Different Species Across Groups】 |
| \| \| \| \| \| \|-- Wilcoxon\_sig.xls | 【Differential Species (p < 0.05) Among Groups】 |
| \| \| \| \| \| \|-- Wilcoxon\_topg\*.xls | 【Top 10 Differential Species by Abundance at Each Taxonomic Level】 |
| \| \| \| \| \| \|-- Wilcoxon\_top\_releative.xls | 【Top 10 Most Abundant Species Table at Each Taxonomic Level】 |
| \| \| \| \| \| \|-- wilcoxonbarplot.{pdf ,png} | 【Mean Abundance Bar Plot Showing Wilcoxon Test Results】 |
| \| \| \| \| \| \|-- wilcoxon\_boxplot.{pdf,png} | 【Mean Abundance Boxplot with Wilcoxon Test Results】 |
| \| \|-- metagenomeseq | 【Metagenomeseq analysis】 |
| \| \| \|-- group1/... | 【Metagenomeseq (Samples in the group are greater than 2)】 |
| \| \| \| \|-- phylum/... | 【Metagenomeseq analysis at each taxonomic level (total,k,p,c,o,f,g,s)】 |
| \| \| \| \| \|-- diffHeatmap | 【Heatmap of top10】 |
| \| \| \| \| \| \|-- annotation.heatmap.{pdf,png} | 【Heatmap analysis results of top10 abundance of species with significant differences】 |
| \| \| \| \| \|-- \*-\* | 【Metagenomeseq for two groups】 |
| \| \| \| \| \| \|-- \*.psig.xls | 【Metagenomeseq results with p value less than 0.05 at each taxonomic level】 |
| \| \| \| \| \| \|-- \*.qsig.xls | 【Metagenomeseq results with q value less than 0.05 at each taxonomic level】 |
| \| \| \| \| \| \|-- boxplot | 【Boxplot diagrams with significant difference species】 |
| \| \| \| \| \| \| \|-- \*.{pdf,png} | 【Visualization of Boxplot diagrams】 |
| \| \|-- simper | 【Similarity percentage (Simper)】 |
| \| \| \|-- group1/... | 【Simper (Samples in the group are greater than 2)】 |
| \| \| \| \|-- Rplots.pdf | 【Visualization of Variance Contribution】 |
| \| \| \| \|-- phylum/... | 【Simper analysis at each taxonomic level (total,k,p,c,o,f,g,s)】 |
| \| \| \| \| \|-- \*-\* | 【Analysis of contribution of top10 species to sample difference between groups】 |
| \| \| \| \| \| \|-- \*-\*.{xls,pdf,png} | 【Analysis of contribution of top10 species to sample difference between groups】 |
| \| \|-- ttest | 【T-test analysis】 |
| \| \| \|-- group1/... | 【T-test (Samples in the group are greater than 2)】 |
| \| \| \| \|-- phylum/... | 【T-test analysis at each taxonomic level (total,k,p,c,o,f,g,s)】 |
| \| \| \| \| \|-- \*-\* | 【Species analysis with significant difference between groups】 |
| \| \| \| \| \| \|-- \*.psig.xls | 【T-test results with p value less than 0.05 at each taxonomic level】 |
| \| \| \| \| \| \|-- \*.qsig.xls | 【T-test results with q value less than 0.05 at each taxonomic level】 |
| \| \| \| \| \| \|-- \*.Volcano.{pdf,png} | 【Volcano diagrams with significant difference species】 |
| \| \| \| \| \| \|-- \*.Volcano.label.{pdf,png} | 【Volcano diagrams with significant difference species (label)】 |
| \| \| \| \| \| \|-- boxplot | 【Boxplot diagrams with significant difference species】 |
| \| \| \| \| \| \| \|-- \*.{pdf,png} | 【Visualization of Boxplot diagrams】 |
| \| \|-- lefse | 【Linear Discriminant Analysis (LDA) effect size analysis】 |
| \| \| \|-- group1/... | 【LEFse (Samples in the group are greater than 2)】 |
| \| \| \| \|-- \*-\* | 【Analysis of LEfSe】 |
| \| \| \| \| \|-- LDA.\*.all.res | 【Statistics results of LDA analysis】 |
| \| \| \| \| \|-- LDA.\*.draw.res | 【Draw results of LDA analysis】 |
| \| \| \| \| \|-- LDA.\*.{svg,png} | 【Barplot of LDA scores】 |
| \| \| \| \| \|-- LDA.\*.tree.{svg,png} | 【Cladogram of LDA scores】 |
| \|-- 10.CommAssembly | 【Community Assembly Mechanism Analysis】 |
| \| \|--bNTI | 【βNTI Analysis】 |
| \| \| \|-- group1/... | 【Grouped βNTI Analysis Results 】 |
| \| \| \| \|-- \*-\* | 【Grouped βNTI Analysis Results】 |
| \| \| \| \|-- bNTI.summary.xls | 【Statistical Summary of Grouped βNTI Results】 |
| \| \| \| \|-- bNTI.xls | 【Pairwise βNTI Values for All Samples】 |
| \| \| \| \|-- bNTI.boxplot.pdf | 【Boxplot Visualization of βNTI Results in PDF Format】 |
| \| \| \| \|-- bNTI.boxplot.png | 【Boxplot Visualization of βNTI Results in PNG Format】 |
| \| \|--nst | 【NST Analysis】 |
| \| \| \|-- group1/... | 【Group-wise Taxonomic NST Analysis 】 |
| \| \| \| \|-- \*-\* | 【Group-wise Taxonomic NST Analysis】 |
| \| \| \| \|-- tNST.summary.xls | 【Summary Statistics of tNST Analysis Results】 |
| \| \| \| \|-- tNST.pairwise.xls | 【Pairwise Results of tNST Analysis】 |
| \| \| \| \|-- tNST.boxplot.pdf | 【Boxplot of tNST Analysis Results (PDF)】 |
| \| \| \| \|-- tNST.boxplot.png | 【Boxplot of tNST Analysis Results (PNG)】 |
| \| \| \| \|-- tNST.PERMANOVA.xls | 【PERMANOVA Test for tNST Analysis】 |
| \| \| \| \|-- tNST.boot.summary.xls | 【Summary Statistics of tNST Bootstrapping Results】 |
| \| \| \| \|-- tNST.boot.compare.xls | 【Group-wise Comparison of tNST Bootstrapping Results】 |
| \| \| \| \|-- tNST.boxplot.pdf | 【Violin & Dot Plot Visualization of Bootstrapping Results (PDF)】 |
| \| \| \| \|-- tNST.boxplot.png | 【Violin & Dot Plot Visualization of Bootstrapping Results (PNG)】 |
| \| \| \| \|-- tNST.concatenate.pdf | 【Combined Boxplot & Violin Plot (PDF)】 |
| \| \| \| \|-- tNST.concatenate.png | 【Combined Boxplot & Violin Plot (PNG)】 |
| \| \|--pNST | 【Group-wise Phylogenetic pNST Analysis】 |
| \| \| \|-- group1/... | 【Group-wise Phylogenetic pNST Analysis 】 |
| \| \| \| \|-- \*-\* | 【Group-wise Phylogenetic pNST Analysis】 |
| \| \| \| \|-- pNST.summary.xls | 【Summary Statistics of pNST Analysis Results】 |
| \| \| \| \|-- pNST.pairwise.xls | 【Pairwise Results of pNST Analysis】 |
| \| \| \| \|-- pNST.boxplot.pdf | 【Boxplot of pNST Analysis Results (PDF)】 |
| \| \| \| \|-- pNST.boxplot.png | 【Boxplot of pNST Analysis Results (PNG)】 |
| \| \| \| \|-- pNST.PERMANOVA.xls | 【PERMANOVA Test for pNST Analysis】 |
| \| \| \| \|-- pNST.boot.summary.xls | 【Summary Statistics of pNST Bootstrapping Results】 |
| \| \| \| \|-- pNST.boot.compare.xls | 【Group-wise Comparison of pNST Bootstrapping Results】 |
| \| \| \| \|-- pNST.boxplot.pdf | 【Violin & Dot Plot Visualization of Bootstrapping Results (PDF)】 |
| \| \| \| \|-- pNST.boxplot.png | 【Violin & Dot Plot Visualization of Bootstrapping Results (PNG)】 |
| \| \| \| \|-- pNST.concatenate.pdf | 【Combined Boxplot & Violin Plot (PDF)】 |
| \| \| \| \|-- pNST.concatenate.png | 【Combined Boxplot & Violin Plot (PNG)】 |
| \| \|--iCAMP | 【Group-wise Phylogenetic iCAMP Analysis】 |
| \| \| \|-- group1/... | 【Group-wise Phylogenetic iCAMP Analysis 】 |
| \| \| \| \|-- \*-\* | 【Group-wise Phylogenetic iCAMP Analysis】 |
| \| \| \| \|-- icamp.ProcessImportance\_EachGroup.xls | 【 Relative Importance of Assembly Processes in Each Group】 |
| \| \| \| \|-- icamp.ProcessImportance\_EachTurnover.xls | 【Relative Importance of Assembly Processes Between Sample Pairs Within Groups Column Descripti】 |
| \| \| \| \|-- icamp.circlexxgroup.pdf | 【 Circular Plot (PDF) Visualizing the Relative Importance of Assembly Processes in Each Group】 |
| \| \| \| \|-- icamp.circle\_{group}\_Group.png | 【Circular Plot (PNG) Visualizing the Relative Importance of Assembly Processes in Each Group】 |
| \| \| \| \|-- icamp.barEachGroup.pdf | 【Bar Plot (PDF) Visualizing the Relative Importance of Assembly Processes Across Groups】 |
| \| \| \| \|-- icamp.barEachGroup.png | 【Bar Plot (PNG) Visualizing the Relative Importance of Assembly Processes Across Groups】 |
| \|-- Advanced\_ReadMe\_EN.html |  |

`Tips:`

- Metagenomeseq, Adonis, MRPP, Anosim, T-Test, LEfSe, KruskalWallis, Wilcoxon and other inter group difference analysis, and the corresponding statistical test can be conducted when each grouping is no less than three samples (if there are no different species between groups, the corresponding test results will not be displayed).
- Community assembly analysis requires the presence of groups with no less than three biological replicates; otherwise, this analysis is not available.
- Random forest analysis requires each group to have no less than 15 biological replicates; otherwise, this analysis is not available.
- The functional prediction and environmental factor sections need to be filled in the collection table. If relevant analyses are not completed, corresponding results will not be displayed.
- Network analysis, the chart can only be generated when the default number of samples is not less than six.

  This directory displays all deliverable analysis results; if there are no results, this file cannot be linked to the corresponding file.

  ​

---

```
```
xxxxxxxxxx
```


```
                           If you have any questions, please consult the project sales or operation.
```
```
